# Pharmacological treatments of presbyopia: a review of modern perspectives

**DOI:** 10.1186/s40662-017-0068-8

**Published:** 2017-02-07

**Authors:** Antonio Renna, Jorge Luciano Alió, Luis Felipe Vejarano

**Affiliations:** 1 Vissum Alicante, Alicante, Spain; 2 Studi Medici Renna, Melendugno Lecce, Italy; 3 Universidad Miguel Hernandez, Calle Cabañal 1, 03016 Alicante, Spain; 4 Vissum Instituto Oftalmologico de Alicante, Alicante, Spain; 5 Fundación Oftalmológica Vejarano, Popayan, Colombia; 6 Universidad del Cauca, Popayan, Colombia

**Keywords:** Presbyopia, Ageing, Near vision, Glasses, Multifocality, Accommodation, Pharmacological therapy, Eyedrops

## Abstract

**Introduction:**

Presbyopia affects people from the 4^th^ decade of life and is characterized by accommodative loss that leads to negative effects on vision-targeted health-related quality of life. A non-invasive pharmacological treatment providing near-lenses independence would be a truly groundbreaking approach in the treatment of presbyopia. The purpose of this review is to analyze the emerging pharmacological solutions proposed to address presbyopia.

**Results:**

Several ophthalmic eye drops compounds solutions have been described in peer-reviewed papers or presented in ophthalmological tabloids and congresses. Each topical treatment deals with drug combinations aimed to modify one or more factors involved in the accommodative process and have been proposed to be instilled either monocularly or binocularly. It remains unclear how much each drug in the final combined form is involved in the achievement of the outcome and contributes to it.

**Conclusion:**

Despite the lack of a completely well understood mechanism, pharmacological control of presbyopia seems to be a possible and very attractive alternative for presbyopic patients. The studies mentioned in this review are to be considered pilot investigations as they involve either a small number of subjects or are single case series. Complete studies are needed to confirm which will be the more effective pharmacological compound for the treatment of presbyopia.

## Background

Presbyopia affects people from the 4^th^ decade of life and is characterized by accommodative loss that leads to negative effects on vision-targeted health-related quality of life [1]. Despite the recent advances in diagnostic tools, the exact role of each factor (hardening of the lens, changes in the elasticity of the lens capsule, lens dimension, geometry of zonular attachments and ciliary muscle contraction) in contributing to the accommodative loss in presbyopia is still debatable. In the last few years, a number of surgical techniques aimed to compensate presbyopia have been proposed, but each one presents some limitations, thus the most recent trends prefer non-surgical solutions for this condition [2]. A non-invasive pharmacological treatment providing near-lenses independence would be a truly groundbreaking approach in the treatment of presbyopia. The purpose of this review is to analyze the current pharmacological solutions proposed to address presbyopia.

## Main text

### Methods

A review on PubMed was performed analyzing all the publications from 2005 to 2016 concerning the topic of the pharmacological treatment of presbyopia (keywords: presbyopia, pharmacological treatment of presbyopia, pharmacological induction of accommodation). Only four papers were found [3–6], underlining the difficulty of finding a topical treatment for presbyopia. As only few papers regarding the pharmacological treatment of presbyopia were found to be published in peer-reviewed journals, data was searched among those that have been presented during international congresses or published in ophthalmological tabloids with an acknowledged Editorial Board scientific surveillance control.

## Results

One of the very few publications on the topic of the pharmacological control of presbyopia was recently published by Abdelkader in 2015. The publication concerns the results of a prospective double-masked randomized placebo-controlled clinical trial involving 48 naturally emmetropic and presbyopic subjects aged between 43 and 56 years, which was aimed at evaluating the efficacy of instilling carbachol 2.25% with brimonidine 0.2% eye drops monocularly once daily for 3 months [3]. The choice of active principles was driven by the rationale of stimulating the parasympathetic innervation and increasing depth of focus through miosis. Accommodation induced by the parasympathomimetic was perhaps associated with the prolongation and potentiation of these effects by the alpha agonist. The results showed a 4-line mean improvement of uncorrected near visual acuity (UNVA) measured with the Jaeger scale 1 h after the instillation of the drops that progressively regressed to 1 to 2 line at 10-hour, without a worsening of the uncorrected far visual acuity (UDVA) at any time or any serious side effects observed. Mild burning sensation was noted by 1 subject (3.3%), dull headache was reported in 10% of the all subjects, and temporary difficulty in low luminosity (dimness) for the first couple of weeks was reported by 1 subject (3.3%). All the 30 subjects in the treated group abandoned the use of near glasses, while receiving treatment, showing satisfaction with both near and distance vision, 12 of these patients (40%) reported that the effect was excellent for the first 8 h and then gradually faded.

An approach using a topically applied pharmacological compound bilaterally was described by Renna et al. in 2016 that showed the results of a prospective pilot study involving 14 presbyopic subjects aged 41 to 55 years [4]. The subject of the report was a study designed and performed by Dr. Vejarano with a patent pending pharmacological combination of active principles (European Patent Application No. EP 13 745 508.5; China Patent Application No. PW34087KMOB) to be instilled binocularly: Pilocarpine 0.247%, Phenylephrine 0.78%, Polyethyleneglycol 0.09%, Nepafenac 0.023%, Pheniramine 0.034%, and Naphazoline 0.003%. The rationale of choice for this combination is because it stimulates the contraction of the ciliary body while maintaining a physiological pupil diameter variation avoiding a worsening of visual performance in dimness condition that would allow a physiological image merging with clear focus at near, intermediate, and long distance. Pilocarpine stimulates accommodation providing both miosis and ciliary body contraction and may improve tear production by stimulating lacrimal gland secretion. Phenylephrine, Nepafenac, and Pheniramine counteract ciliary muscle spasm, vascular congestion and hyperemia induced by pilocarpine, avoiding an excess of pupil constriction. Naphazoline increases acetylcholine release and reduces norepinephrine release empowering pilocarpine’s relaxing effect on dilator pupillae and relieving its side effects. The lubricant effect of polyethyleneglycol protects from the burning typically experienced from most of these compounds and improves the tolerance for using the eye drops. The results showed a mean UNVA improvement by about 2 to 3 lines in each eye and binocularly from a baseline mean of about J 3.5 to about J 1.5, with an improvement ≥ 3 lines until 5 h for seven patients (50% of the total). No patient had a loss in UDVA in each eye and binocularly. All patients enjoyed the near vision that they had after instilling the eye drops and they would like to have a new drop of it to continue the benefits observed. No adverse effects were reported.

C. Feinbaum presented at the 2015 meeting of the American Society of Cataract and Refractive Surgery an investigational proprietary product (PresbiDrops, FEPASAET Group) that combines a parasympathomimetic agent with an NSAID in an oil-based formulation [7]. The rationale is the same with the eye drops studied in the previously mentioned study by Abdekalder. Feinbaum’s study analyzed 81 patients from 42 to 74 years. Ten eyes were pseudophakic, four eyes had cataract, ten eyes were postLASIK or PRK, and 57 were presbyopic without lens opacity. Spherical refraction for the group ranged from −0.75 D to +1.50 D and astigmatism was up to −1.75 D. There were claimed significant improvements in both mean UDVA (from 0.932 to 1.141) and UNVA (0.356 to 0.649). The pseudophakic group showed significant improvements in both UNVA and UDVA, while presbyopic patients who were postrefractive surgery maintained 20/20 distance UDVA and had a significant improvement in UNVA from 0.4 to 0.7. Three-fourths of the patients experienced no adverse reaction. Four patients developed nausea immediately after instillation that quickly resolved, and four patients developed headache that gradually disappeared (duration 10–15 min). Local adverse events included two cases each of dryness or burning, four cases of stinging, and four cases of blurry distance vision, all of which dissipated over 5 min.

The approach proposed by the company, Presbyopia Therapies, to address presbyopia is a drop known as Liquid Vision, binocularly instilled, that has a pure miotic effect without stimulation of accommodation [8]. The rationale is to produce a pinhole effect avoiding the ciliary muscle contraction that would cause a miopic shift worsening far vision. The exact constituents of the drop are proprietary in nature. The company claims a rapid effect on the pupil that leads a stable pupil diameter of approximately 1.6 mm, with a duration of action of approximately 8 h. In the preliminary trials performed by Dr. Castillejos in Tijuana, Mexico, which included patients ranging in age from 46 to 63, near visual acuity improved 3 to 7 lines on the Jaeger scale without compromising distance vision. Patients have reported conjunctival injection upon instillation as well as some stinging. Some minimal dimming indoors is possible for the first few days of use, although patients describe this effect as limited to those first few days. A phase 2 U.S. clinical trial is in the planning stage.

The J. Benozzi method uses pilocarpine 1% and diclofenac 0.1% [5]. The rationale is the same as discussed for the eye drops containing a parasympathomimetic with a nonsteroidal anti-inflammatory drug (NSAID). Benozzi treated 100 patients between 45 and 50 years with his method for 5 years. The author reported that all of patients undertaking the treatment under his survey were showing a near vision of J1 and a far vision of 20/20 instilling the eye drop at 6-hour intervals, daily. Twenty subjects had ocular burning and discomfort after drop instillation, with one of them abandoning treatment because of that. Four other patients abandoned the pharmacological treatment because they feared chronic drop instillation.

Presbyeyedrops© is another eye drop based on a combination of two parasympathomimetics and a NSAID [9]. It was used in a pilot study involving 15 eyes and the authors claimed that they had improvements of UDVA from 0.8 to 1.0 and of UNVA from 0.54 to 0.8. One patient reported nausea after the instillation, one patient reported dryness, and one patient had a burning feeling.

Another eye drop that stimulates the ciliary muscle to accommodate and constricts the pupil is based on the proprietary components of PresbyPlus: two parasympathomimetics and one parasympatholytic [10]. These eye drops were instilled bilaterally twice a day in a clinical trial that showed that 90% of subjects could see J4 to J1 within 1 year without adverse reactions.

A completely different approach for the pharmacological treatment of presbyopia consists in targeting the crystalline lens. A lipoic acid patented derivative drop was developed by Encore Vision to reduce crystalline protein disulfide bonds, aiming to soften the lens, thus preserving the natural shape-changing ability in order to restore the accommodative amplitude [6]. In 2016, the company begun a 90-day phase 1/2 clinical trial including 66 presbyopic subjects between the ages of 45 and 55 years.

## Conclusion

The pharmacological control of presbyopia is a very attractive option for those affected by presbyopia and increasing near vision spectacle dependence. Despite the interest on this topic, there are only a few publications available, all from the recent years. As a non-invasive solution for addressing this problem, pharmacological control of presbyopia would meet all of the established criteria for the severity of presbyopia in different subjects. The pharmacological compounds analyzed in this review aim to target one or more factors involved in the near vision process. Most of the topical products use pharmacological compounds, including a combination of different drugs. Therefore, it remains unclear how much each of the drug in the final combined form is involved in the outcome and contributes to it.

The pharmacological control of presbyopia presents itself, on this review, as a possible and very attractive alternative for presbyopic patients. The studies mentioned in this review are to be considered pilot investigations as they involve either a small number of subjects or are single case series. Moreover, reports presented at international meetings and published on scientific tabloids, are not peer-reviewed. Due to its large interest and potential general application, further and more complete studies are needed to confirm which will be the more effective pharmacological drug for presbyopia treatment. Despite the limitations of the papers reviewed, such preliminary results speak to the possibility of a pharmaceutical treatment for presbyopia. Patient studies are very expensive and probably limited the scope of these investigations (ad hoc patients and private funds).

## Abbreviations

NSAID: Nonsteroidal anti-inflammatory drug
UDVA: Uncorrected far visual acuity
UNVA: Uncorrected near visual acuity

## References

[1] McDonnell PJ, Lee P, Spritzer K, Lindblad AS, Hays RD (2003). Associations of presbyopia with vision-targeted health-related quality of life. Arch Ophthalmol.

[2] Charman WN (2014). Developments in the correction of presbyopia II: surgical approaches. Ophthalmic Physiol Opt.

[3] Abdelkader A (2015). Improved presbyopic vision with miotics. Eye Contact Lens.

[4] Renna A, Vejarano LF, De la Cruz E, Alió JL (2016). Pharmacological treatment of presbyopia by novel binocularly instilled eye drops: a pilot study. Ophthalmol Ther.

[5] Benozzi J, Benozzi G, Orman B (2012). Presbyopia: a new potential pharmacological treatment. Med Hypothesis Discov Innov Ophthalmol.

[6] Crawford KS, Garner WH, Burns W (2014). Dioptin™: A novel pharmaceutical formulation for restoration of accommodation in presbyopes. Invest Ophthalmol Vis Sci.

[7] Krader CG, Feinbaum C (2015). Simple solution for presbyopia: topical agent acts by reducing pupil size to increase depth of focus. Ophthalmology times.

[8] Kim T (2014). A unique drop. Eyeworld.

[9] Patel S, Salamun F, Matovic K. Pharmacological correction of presbyopia. Poster presented at the XXXI congress of the ESCRS. 2013. Amsterdam. http://escrs.org/amsterdam2013/programme/posters-details.asp?id=19804. Accessed 15 Sept 2016.

[10] Donofrio Angelucci D, Pinelli R, Vejarano LF (2016). Presbyopia Eye Drop Targets Miosis and Accommodation. Refractive Surgery Outlook.

