# Erratum to: Pharmacological treatments of presbyopia: a review of modern perspectives

**DOI:** 10.1186/s40662-017-0074-x

**Published:** 2017-03-23

**Authors:** Antonio Renna, Jorge L. Alió, Luis Felipe Vejarano

**Affiliations:** 1 Vissum Alicante, Alicante, Spain; 2 Studi Medici Renna, Lecce, Melendugno, Italy; 3 Universidad Miguel Hernandez, Calle Cabañal 1, Alicante, 03016 Spain; 4 Vissum Instituto Oftalmologico de Alicante, Alicante, Spain; 5 Fundación Oftalmológica Vejarano, Popayan, Colombia; 6 Universidad del Cauca, Popayan, Colombia

## Erratum

After publication of the original article [1] it was brought to our attention that author Jorge L. Alió was incorrectly included as Jorge Luciano Alió. The correct name is included in the author list of this erratum and has been updated in the original article.

## References

[1] Renna A (2017). Pharmacological treatments of presbyopia: a review of modern perspectives. Eye Vis.

